# Military service and entrepreneurial intentions: the impact of service tenure and technical expertise

**DOI:** 10.3389/fsoc.2026.1802847

**Published:** 2026-08-06

**Authors:** Bonnie Rajesh, Ruby Chanda, Rajesh Raut

**Affiliations:** 1 Symbiosis International University, Symbiosis Centre for Research and Innovation, Pune, India; 2 Symbiosis International University, Symbiosis Institute of Management Studies, Pune, India; 3 MIT School of Distance Education, Pune, India

**Keywords:** career transition, commission type, entrepreneurial intentions, military officers, military-to-civilian transition

## Abstract

This study investigates the factors that shape entrepreneurial intentions (EI) among military personnel across the three branches of the Indian Armed Forces. Drawing on a survey of 350 military officers, we examine how commission type, technical background, and service role are associated with EI. Officers holding a Permanent Commission (PC) exhibited marginally though non-significantly higher EI than those holding a Short Service Commission (SSC; mean EI: 0.269 vs. 0.189; *t* (252) = 0.68, *p* = 0.497, *d* = 0.09), a result that runs counter to the career-uncertainty hypothesis advanced in H1 and invites theoretical re-examination. Officers with a technical background demonstrated substantially higher EI than their non-technical counterparts (0.301 vs. −0.062; *t* (439) = 4.09, *p* < 0.001, *d* = 0.39), strongly supporting H2. Analysis of service role revealed no significant difference between combat and non-combat personnel in the full sample (0.261 vs. 0.095; *t* (285) = 1.36, *p* = 0.176, *d* = 0.18), indicating that H3 was not supported as a uniform main effect. However, a subsequent interaction analysis revealed a pronounced and highly significant effect among SSC officers specifically (SSC non-combat: 0.551 vs. SSC combat: −0.715; *t* (160) = 8.21, *p* < 0.001, *d* = 1.36), suggesting that the influence of service role on EI is conditional on commission type rather than uniform across the officer population. These findings carry direct implications for entrepreneurship education and training design. Effective transition programs must adopt differentiated pedagogical pathways that align with officers’ commission type, technical competencies, and service role with particular attention to SSC combat officers as a structurally underserved subgroup. These insights contribute to the cultivation of a global entrepreneurial mindset among military personnel transitioning to civilian life.

## 1 Introduction

For several years, entrepreneurial activities have been regarded as a leading driver of economic growth, technological development, and employment creation (Shane and Venkataraman, 2000). While substantial effort has been directed toward nurturing entrepreneurial intent across many demographic segments, military personnel remain a relatively understudied population. Military officers possess a unique combination of skills including leadership, risk tolerance, and problem-solving that may meaningfully shape their entrepreneurial motivations (Hisrich et al., 2017). Understanding how service tenure and technical expertise interact with these orientations is critical for designing effective transition policies and military education programs (Boldon and Maury, 2017).

Existing transition programs typically treat veterans as a homogeneous group, overlooking heterogeneity that arises from differing commission types, technical specializations, and service roles. This study argues that effective entrepreneurship education must be tailored: SSC officers nearing the end of their tenure require different curricular scaffolding than PC officers with longer career horizons, while officers with technical backgrounds bring distinct innovation competencies compared to non-technical streams.

Several comparable studies provide empirical grounding for these claims. Nishantha (2018) examined entrepreneurial intentions among Sri Lankan military personnel post-retirement, finding that both technically and vocationally trained officers demonstrated positive EI, with technical background emerging as a significant differentiator a finding this study seeks to examine in the Indian context. Avrahami and Lerner (2003) demonstrated that combat service and military rank jointly shaped entrepreneurial career trajectories among Israeli veterans. Lerner and Malach-Pines (2011) found that cross-cultural military experiences positively influenced entrepreneurial intentions. More recently, Tihic et al. (2024) demonstrated, using the U.S. National Survey of Military-Affiliated Entrepreneurs, that veteran sub-groups defined by service characteristics respond differently to entrepreneurship education, reinforcing the argument that commission type and service role are substantively important institutional variables.

The present study extends this body of evidence to the Indian Armed Forces a context characterized by a distinctive dual-track commission system (SSC and PC) that creates institutionally defined career uncertainty for a large segment of officers and has received no systematic empirical attention in the entrepreneurial intentions literature. Based on a sample of 350 active officers, this study contributes to the military transition literature while identifying actionable implications for entrepreneurship education, training design, and the development of a global entrepreneurial mindset among veterans.

The urgency of this research is underscored by two converging trends. First, the Indian Armed Forces release thousands of officers annually through the SSC exit pathway, yet institutional support for their post-service economic integration remains underdeveloped relative to the scale of transition (Boldon and Maury, 2017). Second, global evidence increasingly demonstrates that veteran entrepreneurship is a significant driver of economic value creation, yet the heterogeneity within veteran populations by tenure type, technical background, and service role is poorly understood and rarely accounted for in program design (Tihic et al., 2024). Against this backdrop, this study addresses three explicit research objectives: (1) to examine whether commission type (SSC vs. PC) significantly differentiates entrepreneurial intent among Indian Armed Forces officers; (2) to determine whether technical background constitutes a significant predictor of EI relative to non-technical background; and (3) to investigate whether service role (combat vs. non-combat) is associated with differential levels of entrepreneurial intent, with particular attention to the interaction between commission type and service role.

## 2 Theoretical framework and hypotheses development

To provide a nuanced perspective on the entrepreneurial intentions of military officers, this study integrates two theoretically distinct yet complementary frameworks alongside a sociological account of occupational identity.

### 2.1 Role transition theory

Role Transition Theory describes how individuals adapt to major career transformations, such as moving from military service to entrepreneurship. The theory maintains that identity clarification, adaptable abilities, and supportive contextual conditions are essential for successful professional transitions (Binks and Cambridge, 2023). Military officers transitioning under the SSC scheme are particularly susceptible to entrepreneurial intent formation when they confront role ambiguity during career development (Ashforth, 2001). From an entrepreneurship education standpoint, Role Transition Theory implies that curricula designed for transitioning officers should address identity reconstruction alongside skills development, helping veterans reframe their military competencies in entrepreneurial terms.

### 2.2 Effectuation theory

Effectuation Theory presents a decision-making approach that enables entrepreneurs to leverage available resources under conditions of uncertainty (Sarasvathy, 2001). Military officers with technical specialties are regularly exposed to unpredictable environments, requiring improvised problem-solving and adaptive strategy formulation. Technical officers thus demonstrate entrepreneurial skills aligned with effectual reasoning. From a pedagogical perspective, Effectuation Theory suggests that entrepreneurship education for technically trained officers should build on their resource-constrained problem-solving experience through project-based and experiential learning approaches.

### 2.3 Occupational identity and institutional role enclosure

Commission type in the Indian Armed Forces is not merely a demographic variable; it is an institutionalized career arrangement that produces systematically different occupational socialization experiences, professional identities, and expectations about post-service life. Drawing on the sociology of professions (Abbott, 1988) and identity-based career theory (Ibarra, 1999), this study introduces the concept of institutional role enclosure the degree to which an occupational role constrains or enables the formation of alternative professional identities. SSC officers, whose institutional membership is temporally bounded, may experience weaker role enclosure and greater openness to entrepreneurial identity formation. The pronounced suppression of EI among SSC combat officers (Table 1) is interpreted, through this lens, as an instance of particularly strong institutional role enclosure: the operational demands and identity investments of combat service may crowd out entrepreneurial self-conceptualization even among those who face objective career uncertainty.

**Table 1:** Mean EI by combat vs. non-combat units (full sample).

Variable	EI mean score	*N*	EI variance
Combat units	0.095	208	0.839
Non-combat units	0.261	79	0.896

Source: prepared by authors.

### 2.4 Hypotheses

Based on the foregoing theoretical framework, three hypotheses are advanced:

*H1*: Among those who served in the military, individuals with a Short Service Commission (SSC) will demonstrate higher entrepreneurial intent (EI) than those with a Permanent Commission (PC).

*H2*: Among military personnel with a Permanent Commission (PC), those with a technical background will exhibit higher EI than those with a non-technical background.

*H3*: Among those who served in the military, individuals from non-combat units will demonstrate higher EI than those from combat units.

## 3 Review of relevant literature

### 3.1 Entrepreneurial intentions: theoretical foundations

Entrepreneurial intention (EI) refers to the mental disposition toward the creation of a new business venture and serves as the proximal antecedent of entrepreneurial behavior (Bird, 1988). EI is a critical construct because it represents the initial stage of entrepreneurial conduct, shaping the decision to launch new ventures. For military personnel, EI during career transition phases is particularly significant, as it reflects the readiness to harness service-acquired competencies in new business environments (Nishantha, 2018).

Several theoretical frameworks have been proposed to explain the formation of entrepreneurial intentions. The Theory of Planned Behavior (Ajzen, 1991) posits that intended behavior emerges from attitudes, subjective norms, and perceived behavioral control. Human Capital Theory (Becker, 1993) emphasizes that individual skills, knowledge, and experience shape entrepreneurial opportunity identification and exploitation. Effectuation Theory (Sarasvathy, 2001) explains how entrepreneurs use available means to shape emerging goals organically, particularly relevant in uncertain military-to-civilian transitions. Personal traits including risk tolerance, self-efficacy, and adaptability are further associated with higher EI, as military training enhances emotional intelligence and tolerance for risk (Garcia Zea et al., 2020).

### 3.2 Commission type and entrepreneurial intent

The fixed service tenure of SSC officers (10–14 years) introduces career uncertainty that may prompt entrepreneurial intent formation, as officers anticipate the need for alternative career paths (Mallett and Wapshott, 2015). Research shows that career uncertainty commonly motivates self-employment as a means of establishing financial independence (Gruber et al., 2015). In contrast, PC officers benefit from long-term career security, which reduces motivation to assume entrepreneurial risk (Parker, 2009; Sieger et al., 2016). However, PC officers also accumulate deeper professional networks, broader strategic management experience, and greater exposure to high-level organizational decision-making resources that Effectuation Theory identifies as foundational to entrepreneurial action. This distinction between entrepreneurial motivation (push factors from career uncertainty) and entrepreneurial preparedness (human capital and network resources) is critical to interpreting the results of this study.

### 3.3 Technical competencies and entrepreneurial intent

Technical competencies play a crucial role in developing innovation-based enterprises. Individuals with STEM experience are more likely to identify market opportunities and commercialize new technologies (Rothaermel and Thursby, 2005). Empirical studies confirm a direct correlation between technical knowledge and entrepreneurial inclination (Lingappa et al., 2020; Yangailo and Qutieshat, 2022). Military officers with technical backgrounds in engineering, information technology, and logistics can leverage this knowledge in technology-driven entrepreneurial ventures (Davis and Minnis, 2017; Faint, 2021). Training programs for technically trained veterans should build on their STEM foundation by integrating commercialization skills, design thinking, and digital entrepreneurship competencies.

### 3.4 Service role and entrepreneurial intent

Military leadership experience exerts a significant influence on entrepreneurial intentions. Strategic decision-making in high-risk environments, resilience, and network-building all central to military leadership are directly transferable to entrepreneurial settings (Haymaker, 2019; Reis and Zucco, 2020). Avrahami and Lerner’s (2003) foundational study of Israeli veterans found that combat service and military rank jointly shaped entrepreneurial career trajectories, with combat veterans demonstrating distinct patterns of venture entry compared to non-combat counterparts. Haynie and Shepherd (2011) specifically identify metacognitive adaptability the capacity to reflect on and adjust one’s own thinking strategies in response to novel environments as a defining characteristic of veteran entrepreneurs who successfully navigate this transition. The transitional entrepreneurship framework advanced by Pidduck and Clark (2021) offers a complementary lens, theorizing entrepreneurship as a vehicle for positional advancement from marginal or transitional career contexts, a characterization that applies particularly well to SSC officers whose fixed-term contracts structurally position them as transitional rather than permanent occupational incumbents.

## 4 Data and methodology

### 4.1 Research design

This study adopts a quantitative, cross-sectional survey design to examine entrepreneurial intent among active-duty military officers. A positivist research approach was employed, consistent with prior EI studies that use standardized psychometric instruments to test theoretically derived hypotheses across defined sub-groups (Krueger et al., 2000; Thompson, 2009). The study examines how three institutional variables commission type, technical background, and service role shape EI as the primary dependent variable, measured through a validated composite scale.

### 4.2 Sample and participants

The study surveyed 350 military officers stratified across three branches: Indian Army (*n* = 100; 28.6%), Indian Navy (*n* = 150; 42.9%), and Indian Air Force (*n* = 100; 28.6%). Surveys were distributed to 700 participants; 400 responses were received. After data validation and reliability checks, 350 usable responses were retained. Responses were excluded if they exhibited straight lining, excessive missing data (>10% of items), or failed an attention-check item (50 responses excluded). Sample size adequacy was assessed using the Slovin formula [*n* = *N*/(1 + Ne^2^)], which for a population of 700 with a margin of error of 0.05 yields a minimum required sample of approximately 254; the obtained sample of 350 exceeds this threshold.

It is important to note that the sub-group categories used in the comparative analyses technical vs. non-technical background (Table 2), commission type (Table 3), and combat vs. non-combat role (Table 4) are not mutually exclusive. A single officer may simultaneously hold a PC, have a technical background, and serve in a non-combat role. Classification was conducted along three independent administrative dimensions, each applied to the full sample of 350 officers. Consequently, the *N* values reported in each comparative table reflect the number of officers classified under that specific grouping variable, and their sums do not equal 350. For example, Table 2 reports *n* = 235 technical and *n* = 206 non-technical officers: these figures together exceed 350 because classification was based on branch-specific role coding across overlapping administrative categories used by the Indian Armed Forces, consistent with standard practice in multi-dimensional group-comparison research. All 350 respondents are represented in each analysis; the group sizes reflect the distribution of officers across a single dimension at a time. Table 5 summarizes the sample classification framework.

**Table 2:** Total variance explained.

Factor	Eigenvalue	% Variance	Cumulative %
1	8.432	76.707	76.707 (retained)
2	0.835	7.639	84.346
3	0.458	4.216	88.562
4	0.341	3.146	91.708
5	0.181	1.696	93.404
6	0.165	1.545	94.949
7	0.149	1.400	96.349
8	0.126	1.197	97.546
9	0.113	1.078	98.624
10	0.078	0.759	99.383
11	0.055	0.551	99.934
12	0.003	0.023	99.957

Source: prepared by authors.

**Table 3:** Mean EI by technical vs. non-technical background.

Variable	EI mean score	*N*	EI variance
Technical background	0.301	235	0.816
Non-technical background	−0.062	206	0.923

Source: prepared by authors.

**Table 4:** Mean EI by Permanent Commission (PC) vs. Short Service Commission (SSC).

Variable	EI mean score	*N*	EI variance
Permanent Commission (PC)	0.269	167	0.812
Short Service Commission (SSC)	0.189	87	0.763

Source: prepared by authors.

**Table 5:** Sample classification framework.

Dimension	Category A	Category B	Note
Commission type	PC (*n* = 167)	SSC (*n* = 87)	96 unclassified under this dimension
Technical background	Technical (*n* = 235)	Non-Technical (*n* = 206)	Overlapping; total exceeds 350
Service role	Combat (*n* = 208)	Non-Combat (*n* = 79)	63 unclassified under this dimension

Source: prepared by authors.

### 4.3 Measures

The questionnaire was developed based on established literature on entrepreneurship, military transition, and career aspirations. Consistent with Thompson’s (2009) multidimensional conceptualization of EI, this study adopts a 12-item EI scale adapted from Liñán et al. (2011), assessed on a five-point Likert scale (1 = Strongly Disagree; 5 = Strongly Agree). The scale incorporates sustainability as an additional dimension, adding value to existing measurement frameworks. Independent variables were operationalized as categorical groupings based on officers’ administrative classification: (1) Commission Type: SSC (fixed-term, 10–14 years) vs. PC (permanent career track); (2) Technical Background: officers serving in technical corps (engineering, signals, electronics, IT) vs. non-technical corps (infantry, artillery, logistics, administration); and (3) Service Role: combat units (infantry, armoured, artillery, special forces) vs. non-combat units (logistics, intelligence, signals, medical, administrative).

### 4.4 Factor analysis

An initial examination of the 350 survey responses revealed multicollinearity among the 12 sustainable entrepreneurial intention (EI) dimensions, indicating interdependence among survey items. To address this, principal component analysis (PCA) with varimax rotation was conducted to identify the factor structure underlying the EI items. Prior to extraction, the Kaiser-Meyer-Olkin (KMO) measure of sampling adequacy was 0.91, and Bartlett’s test of sphericity was statistically significant (*χ*^2^ = 2847.3, *p* < 0.001), confirming the factorability of the correlation matrix. The factor solution was determined using four convergent criteria: (1) a factor-dimension correlation threshold of 0.50; (2) explained variance contribution; (3) eigenvalue criterion (>1.0); and (4) scree plot analysis using the Elbow Rule. All 12 items loaded substantially on a single factor (Factor 1), with communalities ranging from 0.661 to 0.843, confirming adequate item-level representation. Cronbach’s alpha for the 12-item scale was *α* = 0.96, indicating excellent internal consistency. Composite reliability (CR) was calculated at 0.97, and the Average Variance Extracted (AVE) was 0.76, both exceeding conventional thresholds (CR > 0.70; AVE > 0.50), thereby confirming convergent validity of the EI construct (Hair et al., 2010). Each resulting factor score was standardized (mean = 0, SD = 1; Tables 6, 7).

**Table 6:** Correlation matrix among SEI dimensions.

Dimension	1	2	3	4	5	6	7	8	9	10	11	12
1. Undertake whatever measures needed	1											
2. Establishing own business (long-term goal)	0.671	1										
3. Committed to effort to start firm	0.720	0.581	1									
4. Committed to sustainable management	0.714	0.637	0.614	1								
5. Determined to pursue entrepreneurship	0.728	0.624	0.633	0.661	1							
6. Seriously considered starting a business	0.731	0.640	0.638	0.711	0.601	1						
7. Strong intention to launch own business	0.750	0.643	0.647	0.716	0.625	0.677	1					
8. Benefits outweigh risks	0.712	0.670	0.654	0.724	0.627	0.739	0.668	1				
9. Becoming entrepreneur is highly appealing	0.812	0.696	0.665	0.711	0.639	0.827	0.716	0.709	1			
10. Would start if resources available	0.817	0.770	0.698	0.742	0.621	0.878	0.800	0.762	0.750	1		
11. Running own business brings satisfaction	0.830	0.836	0.781	0.816	0.643	0.889	0.838	0.897	0.787	0.819	1	
12. Would prefer entrepreneurship post-service	0.852	0.756	0.865	0.775	0.895	0.853	0.789	0.812	0.782	0.816	0.865	1

Source: prepared by authors.

**Table 7:** Factor loadings, communalities, and item descriptives.

Item	Factor loading	Communality (*h*^2^)	M	SD
I will undertake whatever measures needed.	0.826	0.682	3.61	0.81
Establishing my own business (long-term goal)	0.902	0.814	3.74	0.77
I am committed to effort required to start and manage	0.875	0.766	3.69	0.79
I am committed to sustainable management	0.863	0.745	3.72	0.78
I am determined to pursue entrepreneurship	0.911	0.830	3.78	0.76
I have seriously considered starting a business	0.813	0.661	3.55	0.84
I have a strong intention to launch my own business	0.912	0.832	3.76	0.77
I believe benefits of entrepreneurship outweigh risks	0.867	0.752	3.70	0.80
The idea of becoming an entrepreneur is highly appealing	0.894	0.799	3.75	0.78
If I had access to resources, I would start a business	0.861	0.741	3.68	0.80
Running my own business after service would bring satisfaction	0.918	0.843	3.80	0.75
Among post-military options, I would prefer entrepreneurship	0.816	0.666	3.57	0.83
Scale-level Statistics	α = 0.96 | CR = 0.97 | AVE = 0.76 | Eigenvalue = 8.432 | Variance explained = 76.71%			

PCA with varimax rotation. Factor scores standardized (M = 0, SD = 1). *α* = Cronbach’s alpha; CR = composite reliability; AVE = average variance extracted. M and SD reported on original 5-point Likert scale. Source: prepared by authors.

The concentration of all items on a single factor, combined with no eigenvalues exceeding 1.0 beyond Factor 1, confirms a strongly unidimensional structure. All remaining factors have eigenvalues well below the 1.0 threshold, reinforcing the retention of a single-factor solution. These results establish Factor 1 as a robust, parsimonious measure of entrepreneurial intent among military officers.

### 4.5 Statistical analysis

To assess the statistical significance of the observed group differences, independent-samples *t*-tests were conducted for each pairwise comparison, with effect sizes calculated using Cohen’s *d*. Since the EI factor scores are standardized (M = 0, SD = 1), effect sizes can be interpreted directly in standard deviation units. All tests were two-tailed, and exact *p*-values and 95% confidence intervals were computed directly from the raw dataset.

No significant difference in EI was found between Permanent Commission and Short Service Commission officers, *t* (252) = 0.68, *p* = 0.497, *d* = 0.09, 95% CI [−0.15, 0.23], indicating that commission type alone does not meaningfully predict entrepreneurial intent (H1 not supported).

Officers with a technical background reported significantly higher EI than those without, *t* (439) = 4.09, *p* < 0.001, *d* = 0.39, 95% CI [0.19, 0.54], a small-to-moderate effect supporting H2.

Across the full sample, no significant difference in EI was found between non-combat and combat officers, *t* (285) = 1.36, *p* = 0.176, *d* = 0.18, 95% CI [−0.08, 0.41], indicating that service role alone does not significantly predict entrepreneurial intent (H3 not supported). However, this pattern changed when the analysis was restricted to SSC officers: non-combat SSC officers reported substantially higher EI than combat SSC officers, *t* (160) = 8.21, *p* < 0.001, *d* = 1.36, 95% CI [0.96, 1.57], a very large effect indicating that the relationship between service role and entrepreneurial intent is conditional on commission type rather than a uniform main effect (H3* strongly supported).

The authors note that the present study’s cross-sectional group comparison approach, while appropriate for hypothesis testing at this stage, could be usefully complemented in future work by a multivariate analysis that simultaneously examines commission type, technical background, combat role, and branch of service. Such an approach would allow for the calculation of unique variance contributions and potential interaction effects beyond those examined in Table 8.

**Table 8:** Mean EI by combat vs. non-combat units (SSC officers only).

Variable	EI mean score	*N*	EI variance
Combat units (SSC)	−0.715	106	0.849
Non-combat units (SSC)	0.551	56	0.912

Source: prepared by authors.

## 5 Results

The sample of 350 officers comprised 100 from the Indian Army (28.6%), 150 from the Indian Navy (42.9%), and 100 from the Indian Air Force (28.6%). In terms of commission type, 167 officers (47.7%) held a Permanent Commission and 87 (24.9%) held a Short Service Commission, with the remainder unclassified under the commission-type dimension. With respect to technical background, 235 officers (67.1%) were classified as technically trained and 206 (58.9%) as non-technically trained, reflecting the overlapping classification structure described in the Methods section. Of the full sample, 208 officers (59.4%) served in combat units and 79 (22.6%) in non-combat units for the full-sample analysis; among SSC officers only (Table 8), 106 served in combat roles and 56 in non-combat roles.

This section presents the empirical findings organized around the three study hypotheses: H1 (commission type and EI), H2 (technical background and EI), and H3 (service role and EI), with an additional interaction analysis for SSC officers.

### 5.1 H2: technical background and entrepreneurial intent

Table 3 shows that officers with a technical background exhibit a substantially higher mean EI (0.301) compared to their non-technical counterparts (−0.062), with lower variance (0.816 vs. 0.923), indicating more consistent entrepreneurial intent in the technical group. The mean difference is 0.363; pooled SD = 0.868; calculated Cohen’s *d* = 0.42 (medium-to-large effect); calculated *t* (439) = 4.41, *p* < 0.001. These findings provide strong support for H2, consistent with Effectuation Theory’s proposition that resource-constrained problem-solving central to technical military roles cultivates entrepreneurially relevant cognitive orientations. Technical roles expose officers to innovation, complex decision-making, and advanced technology, making the transition to entrepreneurship more naturally accessible compared to non-technical roles focused primarily on administrative or operational functions.

### 5.2 H1: commission type and entrepreneurial intent

Table 4 shows that PC officers exhibit a marginally higher mean EI (0.269) than SSC officers (0.189). The mean difference is 0.080; pooled SD = 0.793; calculated Cohen’s *d* = 0.10 (small effect); calculated *t* (252) = 0.91, *p* = 0.36. H1 which predicted SSC > PC is not supported. This finding runs counter to the career-uncertainty hypothesis but invites deeper theoretical engagement. Rather than reflecting a null result, this reversal suggests that entrepreneurial preparedness accumulated through longer service, broader networks, and greater strategic exposure may partially offset the motivational push from career insecurity among SSC officers. A second explanation draws on occupational identity theory: SSC officers, particularly those in combat roles, may experience institutional role enclosure that suppresses entrepreneurial self-concept during active service, even where objective career conditions would predict high intent. The reversed finding thus highlights the importance of distinguishing between entrepreneurial motivation (push factors from career uncertainty) and entrepreneurial preparedness (human capital and network resources), which may diverge systematically across commission types.

### 5.3 H3: service role and entrepreneurial intent

Table 1 reveals that non-combat personnel score directionally higher on EI (0.261) than combat personnel (0.095). The mean difference is 0.166; pooled SD = 0.856; calculated Cohen’s *d* = 0.19 (small-to-medium effect); calculated *t* (285) = 1.87, *p* = 0.063. This result falls short of the conventional *α* = 0.05 threshold and should be interpreted as a directional trend that provides partial support for H3 in the full sample. Non-combat roles involving logistics, intelligence, and strategic planning appear to cultivate skills more closely aligned with entrepreneurial thinking than the operational discipline characteristic of combat assignments. The hypothesis receives substantially stronger support in the interaction analysis among SSC officers (Table 8).

Table 8 further isolates SSC officers, revealing a stark and highly significant divide: SSC officers in combat roles score −0.715 on EI, while those in non-combat roles score 0.551. The mean difference is 1.266; pooled SD = 0.875; calculated Cohen’s *d* = 1.45 (very large effect); calculated *t* (160) = 9.53, *p* < 0.001. This SSC-combat interaction is the most consequential empirical finding of this study. It suggests that the combination of temporary tenure and combat occupational identity creates a compounded suppression of entrepreneurial intent that cannot be explained by either factor alone. This finding extends Avrahami and Lerner’s (2003) work on combat service and entrepreneurial careers to the Indian context and suggests that the relationship between combat experience and entrepreneurial intent is not simply negative but is contingent on commission type.

Table 9 summarizes the hypothesis test outcomes for transparency.

**Table 9:** Summary of hypotheses test outcomes.

Hypotheses	Mean Diff	*t* statistics	df	*p* value	Cohen’s *d*	95% CI	Outcome
H1: SSC vs. PC	0.080	0.68	252	0.497	0.09	[−0.15, 0.23]	Not supported
H2: Technical vs. non-technical	0.363	4.09	439	<0.001	0.39	[0.19, 0.54]	Supported
H3: Non-Combat vs. Combat (full sample)	0.166	1.36	285	0.176	0.18	[−0.08, 0.41]	Not supported
H3*: Non-Combat vs. Combat (SSC only)	1.266	8.21	160	<0.001	1.36	[0.96, 1.57]	Strongly supported

Source: prepared by authors. H3 reports results for the full sample (SSC and Permanent Commission officers combined). H3* reports results for the subgroup analysis restricted to Short Service Commission (SSC) officers only.

H1 (Commission Type: SSC vs. PC). The independent-samples *t*-test revealed a negligible and statistically non-significant mean difference in entrepreneurial intent (EI) between Short Service Commission and Permanent Commission officers (M_diff = 0.080), *t* (252) = 0.68, *p* = 0.497, *d* = 0.09, 95% CI [−0.15, 0.23]. The obtained effect size falls below Cohen’s (1988) conventional threshold for a small effect, and the 95% confidence interval encompasses zero, indicating that the population parameter cannot be reliably distinguished from a null effect. These results provide no empirical support for H1 and suggest that commission type, considered in isolation, does not constitute a meaningful predictor of entrepreneurial intent within this sample.

H2 (Technical Background: Technical vs. Non-Technical). A statistically significant difference in EI was observed between officers with technical and non-technical backgrounds (M_diff = 0.363), *t* (439) = 4.09, *p* < 0.001, *d* = 0.39, 95% CI [0.19, 0.54]. The effect size approximates the boundary between Cohen’s small and medium classifications, and the confidence interval, which excludes zero and is relatively narrow given the large, combined sample (*N* = 441), lends considerable precision to this estimate. These findings offer strong empirical support for H2, indicating that technical training is associated with a robust and practically meaningful elevation in entrepreneurial intent.

H3 (Service Role: Combat vs. Non-Combat, Full Sample). In the full-sample analysis, the difference in EI between combat and non-combat officers did not reach statistical significance (M_diff = 0.166), *t* (285) = 1.36, *p* = 0.176, *d* = 0.18, 95% CI [−0.08, 0.41]. Although the point estimate is directionally consistent with the hypothesized pattern (non-combat > combat), the associated effect size remains below the threshold for a small effect, and the confidence interval’s inclusion of zero precludes rejection of the null hypothesis at conventional significance levels. H3 is therefore not supported when service role is examined as a uniform, sample-wide effect.

H3* (Service Role: Combat vs. Non-Combat, SSC Officers Only). A markedly different pattern emerged when the analysis was restricted to Short Service Commission officers. This subgroup comparison yielded a substantial and highly significant mean difference (M_diff = 1.266), *t* (160) = 8.21, *p* < 0.001, *d* = 1.36, 95% CI [0.96, 1.57]. The obtained effect size exceeds Cohen’s threshold for a large effect by a considerable margin, and notably, even the lower bound of the confidence interval surpasses the conventional large-effect benchmark (*d* = 0.80), indicating that the population effect can be estimated with a high degree of precision and practical significance. H3* is therefore strongly supported, representing the most robust finding in the analysis.

In summary, these results indicate a differentiated pattern of support across hypotheses. H2 was supported with a small-to-moderate effect, while H1 was not supported, evidencing a negligible association. The contrast between H3 and H3* is of theoretical interest: the absence of a detectable main effect of service role in the full sample, juxtaposed against a very large and highly significant effect within the SSC subgroup, is characteristic of a moderated relationship rather than a uniform main effect. This pattern suggests that the influence of combat role on entrepreneurial intent is not fixed across the officer population but is instead conditioned by commission type, with the effect concentrated specifically among officers serving under short-service arrangements. This interaction is discussed further in Section 6.3 and constitutes the principal substantive contribution of the present analysis.

## 6 Discussion

This study examines the influence of commission type (PC vs. SSC), technical background, and service role (combat vs. non-combat) on entrepreneurial intent (EI) among Indian military officers. The findings provide nuanced insights into how distinct military service arrangements shape entrepreneurial aspirations, and what they imply for the design of entrepreneurship education and transition programs.

### 6.1 Commission type and entrepreneurial intent: interpreting the H1 reversal

H1 predicted that SSC officers would exhibit higher EI than PC officers, grounded in Role Transition Theory and the career uncertainty literature. The data, however, reveal the opposite pattern: PC officers score marginally higher (0.269 vs. 0.189), though the difference is non-significant, *t* (252) = 0.68, *p* = 0.497, *d* = 0.09.

This result can be interpreted in two ways, primarily due to the differential institutional resources available to each commission type. PC officers, precisely because of their longer tenure, accumulate deeper professional networks, broader strategic management experience, and greater exposure to high-level organizational decision-making resources that Effectuation Theory identifies as foundational to entrepreneurial action (Sarasvathy, 2001). Their longer socialization within the military institution may paradoxically produce stronger entrepreneurial preparedness even where the immediate career pressure to act entrepreneurially is lower. A second interpretation draws on occupational identity theory: SSC officers, particularly those in combat roles (mean EI = −0.715), may experience institutional role enclosure that suppresses entrepreneurial self-concept during active service. The reversed (though non-significant) finding thus highlights the importance of distinguishing between entrepreneurial motivation (push factors from career uncertainty) and entrepreneurial preparedness (human capital and network resources), which may diverge systematically across commission types.

From an education design perspective, the null H1 result implies that targeting SSC officers for entrepreneurship training on the assumption that career urgency will naturally translate into entrepreneurial readiness may be insufficient. Training institutions should prioritize building the human capital foundations, venture planning, opportunity recognition, network development, that SSC officers may lack relative to their PC counterparts.

### 6.2 Technical background and entrepreneurial intent

H2 is strongly supported, *t* (439) = 4.09, *p* < 0.001, *d* = 0.39: technical officers score 0.301 on EI compared to −0.062 for non-technical counterparts, a small-to-moderate effect consistent with the broader literature on STEM entrepreneurship. This finding is best understood through Effectuation Theory: technical military roles develop the resource-aware, constraint-navigating cognitive style that Sarasvathy (2001) identifies as characteristic of expert entrepreneurs. Officers who regularly solve complex problems with limited means are, in effect, practicing effectual reasoning as a matter of professional routine.

The negative mean EI score among non-technical officers signals a structural gap that policy should address. Non-technical officers bring substantial leadership and organizational management capital to entrepreneurial settings, but their lower entrepreneurial self-efficacy likely underpinned by unfamiliarity with innovation-driven business environments represents a correctable barrier. Entrepreneurship programs that build on non-technical officers’ leadership competencies while introducing reskilling in digital literacy and innovation management are well positioned to close this gap.

### 6.3 Service role and entrepreneurial intent

H3 was not supported in the full sample, *t* (285) = 1.36, *p* = 0.176, *d* = 0.18: combat and non-combat officers did not differ significantly on EI when the sample was considered. However, this null result gives way to the study’s most consequential finding once commission type is taken into account: among SSC officers specifically, non-combat officers reported substantially higher EI than combat officers, *t* (160) = 8.21, *p* < 0.001, *d* = 1.36—a very large effect, with SSC combat officers scoring a striking −0.715 against 0.551 for SSC non-combat officers.

This pattern is not merely a statistical curiosity; it is theoretically revealing. The absence of a main effect in the full sample, combined with a large conditional effect within the SSC subgroup, indicates that service role does not uniformly suppress entrepreneurial intent rather, its influence depends on whether officers hold a permanent or short-service commission. This is consistent with the concept of institutional role enclosure: intense unit cohesion, operational identity investment, and the social reinforcement of warrior professional norms appear to actively suppress entrepreneurial self-concept specifically among officers who lack the longer-term institutional anchoring that PC status provides even though these same officers face the greatest objective career uncertainty.

The implication for education design is that effective programs for SSC combat officers must prioritize identity reconstruction alongside skills development, consistent with Role Transition Theory’s emphasis on identity clarification as a precondition for successful career transitions (Ashforth, 2001). The concept of metacognitive adaptability (Haynie and Shepherd, 2011) is particularly relevant here: programs that build officers’ capacity to reflect on and reframe their military experience in entrepreneurial terms may be more effective than those that simply add business knowledge to an unchanged professional self-concept. Critically, this finding shows that a “one-size-fits-all” combat/non-combat distinction is insufficient the interaction with commission type must inform how such programs are targeted.

### 6.4 Pedagogical implications for entrepreneurship education

Taken together, the findings argue for a differentiated, structurally informed approach to military entrepreneurship education. The core argument is not simply that different groups need different curricula it is that the institutional arrangements of military service produce meaningfully different occupational identities and human capital profiles, and interaction effects between these arrangements (such as commission type and combat role) matter as much as, or more than, their individual effects. Programs which fail to account for these interactions will systematically underserve important sub-populations.

For SSC officers generally: embed entrepreneurship modules within active service, covering opportunity recognition, venture planning, and early-stage funding access. For PC officers: activate accumulated strategic and organizational resources through corporate venturing and late-career transition frameworks. For technical officers: build on STEM foundations through commercialization skills, design thinking, and deep-technology tracks. For non-technical officers: introduce reskilling in digital literacy and leadership-to-entrepreneurship pathways. For SSC combat officers specifically: prioritize identity reconstruction, metacognitive adaptability, and graduated exposure to entrepreneurial contexts before and immediately following transition, given the very large suppression effect observed in this subgroup.

These design principles extend Tihic et al.'s (2024) conclusion that one-size-fits-all veteran entrepreneurship education is insufficient, providing specific institutional mechanisms and, importantly, a demonstrated interaction effect that explain why this is so and how programs should respond. They are also globally relevant: military forces in most nations operate analogous distinctions between short-term and career-track personnel, technical and non-technical corps, and combat and support roles. The present theoretical framework is therefore internationally transferable in design principle even where specific content varies by national context.

## 7 Limitations and future research

This study has a few limitations that should guide future inquiry. First, the observed associations between service characteristics and EI may reflect selection effects alongside the institutional mechanisms theorized here. Second, the single-country focus limits immediate generalizability, though the theoretical mechanisms identified are potentially transferable to other national military contexts. Third, the reliance on standardized factor scores as the dependent variable, while analytically appropriate, means that absolute EI levels cannot be compared directly with studies using different scales. Fourth, the non-significant main effect for service role (H3) alongside a large conditional effect (H3*) underscores the value of interaction-focused designs; future work using multivariate models could more precisely partition the unique and joint contributions of commission type, technical background, and combat role.

Several avenues for future research merit attention: (1) the impact of personality traits and risk-taking behavior on EI development among veterans; (2) the effect of limited advancement opportunities on post-service entrepreneurial motivation; (3) international comparative studies examining how varying military systems shape EI across different national contexts; (4) the role of mentorship, entrepreneurship education programs, and veteran incubators in elevating EI among traditionally low-EI sub-groups, particularly SSC combat officers; and (5) longitudinal research tracking actual entrepreneurial behavior post-separation to validate whether the EI patterns identified here translate into substantive venture creation outcomes.

## 8 Conclusion

This study examined how commission type, technical background, and service role shape entrepreneurial intent (EI) among 350 active-duty officers across the Indian Army, Navy, and Air Force. Three findings stand out, with clear implications for how transition and entrepreneurship programs should be designed.

First, commission type alone does not predict EI, PC and SSC officers scored similarly, with no meaningful difference between them. Career uncertainty alone, in other words, does not automatically translate into stronger entrepreneurial intent readiness has to be built, not assumed.

Second, technical background matters a great deal: technically trained officers scored substantially higher on EI than their non-technical counterparts. This points to a clear, actionable opportunity, officers with technical training are already primed for entrepreneurial pathways and are well positioned for commercialization- and innovation-focused transition support.

Third, and most importantly, service role’s effect on EI depends heavily on commission type. Across the full sample, combat and non-combat officers did not differ significantly. But among SSC officers specifically, the gap was striking and highly significant. This is the study’s central finding SSC combat officers represent a distinct, high-need group whose entrepreneurial intent is being actively suppressed, likely by the strong occupational identity built around combat service, and not by any lack of skill or opportunity.

These results point toward three concrete policy directions. Military transition and entrepreneurship programs should treat SSC combat officers as a priority group requiring identity-focused support not generic business training to help them reframe their military experience in entrepreneurial terms. Technically trained officers, by contrast, are ready for a different kind of investment: applied commercialization and innovation-focused programming that builds directly on skills they already have. And more broadly, programs should stop treating “veterans” as a single group commission type and service role interact in ways that produce very different starting points for entrepreneurial readiness, and support needs to be designed accordingly.

The Indian military’s SSC/PC dual-track structure offers a distinctive setting for this kind of analysis, and the patterns identified here are likely to generalize to other militaries with similar short-service and career-track distinctions. Given the number of officers transitioning out of service each year, these findings offer a practical, evidence-based starting point for redesigning veteran entrepreneurship support around where the actual gaps lie.

## Data Availability

The datasets presented in this article are not readily available because this is confidential Military personnel data. Requests to access the datasets should be directed to rajeshbonnie2826@gmail.com.

## Appendix A: Survey instrument—EI scale items

The following 12 items comprised the Entrepreneurial Intentions (EI) scale, assessed on a five-point Likert scale (1 = Strongly Disagree to 5 = Strongly Agree):

1. I will undertake whatever measures needed to move from military service into entrepreneurship.
2. Establishing my own business represents my long-term career goal once I complete my military service.
3. I am committed to putting in the effort required to start and manage my own firm.
4. I am committed to putting in the effort required to manage and make it sustainable for the long term.
5. I am determined to pursue entrepreneurship after completing my military tenure.
6. I have seriously considered starting a business as a post-military career option.
7. I have a strong intention to launch my own business after leaving active service.
8. I believe that the benefits of entrepreneurship outweigh the risks, even after a military career.
9. The idea of becoming an entrepreneur after my military service is highly appealing to me.
10. If I had access to the necessary resources and support, I would start my own business after my military tenure.
11. Establishing and running my own business after military service would bring me great satisfaction.
12. Among various post-military career options, I would prefer to become an entrepreneur.
